# Glacier Geometry Changes in the Western Shore of Admiralty Bay, King George Island over the Last Decades

**DOI:** 10.3390/s21041532

**Published:** 2021-02-23

**Authors:** Mariusz Pasik, Krzysztof Bakuła, Sebastian Różycki, Wojciech Ostrowski, Maria Elżbieta Kowalska, Anna Fijałkowska, Marcin Rajner, Sławomir Łapiński, Ireneusz Sobota, Marek Kejna, Katarzyna Osińska-Skotak

**Affiliations:** 1 Faculty Geodesy and Cartography, Warsaw University of Technology, Pl. Politechniki 1, 00-661 Warszawa, Poland; mariusz.pasik@pw.edu.pl (M.P.); krzysztof.bakula@pw.edu.pl (K.B.); sebastian.rozycki@pw.edu.pl (S.R.); wojciech.ostrowski@pw.edu.pl (W.O.); maria.kowalska@pw.edu.pl (M.E.K.); anna.fijalkowska@pw.edu.pl (A.F.); marcin.rajner@pw.edu.pl (M.R.); katarzyna.osinska-skotak@pw.edu.pl (K.O.-S.); 2 Faculty of Earth Sciences and Spatial Management, Department of Hydrology and Water Management, Polar Research Center, Nicolaus Copernicus University, Lwowska 1, 87-100 Toruń, Poland; irso@umk.pl; 3 Faculty of Earth Sciences and Spatial Management, Department of Meteorology and Climatology, Nicolaus Copernicus University, Lwowska 1, 87-100 Toruń, Poland; makej@umk.pl

**Keywords:** glacier recession, glacier, glacier elevation change, glacier thickness change, climate change, DEM, image matching, archival images

## Abstract

This paper presents changes in the range and thickness of glaciers in Antarctic Specially Protected Area (ASPA) No. 128 on King George Island in the period 1956–2015. The research indicates an intensification of the glacial retreat process over the last two decades, with the rate depending on the type of glacier front. In the period 2001–2015, the average recession rate of the ice cliffs of the Ecology Glacier and the northern part of the Baranowski Glacier was estimated to be approximately 15–25 m a^−1^ and 10–20 m a^−1^, respectively. Fronts of Sphinx Glacier and the southern part of the Baranowski Glacier, characterized by a gentle descent onto land, show a significantly lower rate of retreat (up to 5–10 m a^−1^ 1). From 2001 to 2013, the glacier thickness in these areas decreased at an average rate of 1.7–2.5 m a^−1^ for the Ecology Glacier and the northern part of the Baranowski Glacier and 0.8–2.5 m a^−1^ for the southern part of the Baranowski Glacier and Sphinx Glacier. The presented deglaciation processes are related to changes of mass balance caused by the rapid temperature increase (1.0 °C since 1948). The work also contains considerations related to the important role of the longitudinal slope of the glacier surface in the connection of the glacier thickness changes and the front recession.

## 1. Introduction

**Figure 1:**
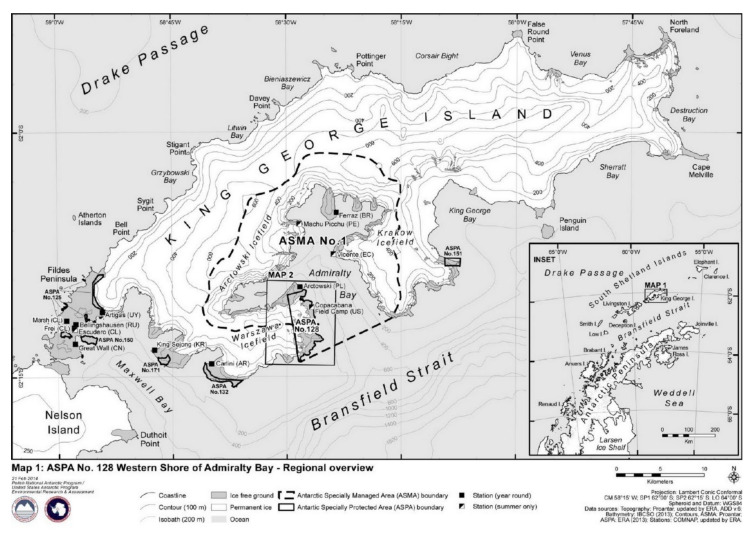
The range and localization of Antarctic Specially Protected Area (ASPA) No. 128 [1].

King George Island belongs to the South Shetland Islands Archipelago situated near the northern edge of the Antarctic Peninsula. At Poland’s request, the of Admiralty Bay was appointed as a Site of Special Scientific Interest (SSSI) No. 8 in 1979. In 2002, the name changed to Antarctic Specially Protected Area (ASPA) No. 128 and, in 2006, the area was incorporated into a newly created Antarctic Specially Managed Area (ASMA) No. 1 Admiralty Bay, King George Island, South Shetland Islands (Figure 1).

The area is very interesting in terms of research because of its clearly visible deglaciation processes. Six glaciers fed by the Warszawa Icefield (Ecology, Sphinx, Baranowski, Tower, Thawing—also known as Dead and Windy) can be found within the ASPA No. 128 boundaries. This study aims to analyze the range and thickness changes for the first three of the listed glaciers over a period of several decades. The selected glaciers represent all types of glaciers occurring in ASPA No. 128.

One of the first pieces of information on the glacier ranges in Admiralty Bay originated in 1962 and was recorded on a British navigation map. The first Polish study of the glacier ranges in this area was carried out during the 2nd Antarctic Expedition (1977–1978) [2]. During the subsequent expedition (1978–1979), aerial photographs were taken that served as the source material for the creation of a topographic map in 1:25000 scale [3], which was the first Polish cartographic study presenting the glacier ranges in the western shore of Admiralty Bay.

In 1983, the first study concerning the changes in the glacier ranges in this area was published [4]. It indicated that the areas were characterized by both intensive recession and distinctive advances when compared with 1961. Later research [5] proved that the glacier retreat was occurring at a high rate, but that this was variable over time. From 1987 to 1989, geodetic surveys of the of Admiralty Bay were carried out by Zbigniew Battke. The results of those measurements were used to create a map in 1:50,000 scale [6], which was also used to illustrate the glacier ranges in ASPA No. 128.

Recession analysis of the Ecology Glacier situated in ASPA No. 128 was also the topic of research by Kejna [7] and Birkenmajer [8]. They covered the periods from 1961 to 1996 and 1956 to 2001, respectively. In 2002, a topographic map of the SSSI No. 8 in 1:12,500 scale presenting, among other features, the glacier ranges and their morphology was created by Pudełko [9,10] using the measurements taken during the 25th Expedition (2000/2001). In 2007, the same author generated an orthophotomap of the Western shore of Admiralty Bay in 1:10,000 scale, based on the previously mentioned aerial photographs taken in 1979 [11,12]. This was the first Polish cartographic study presenting changes in the glacier ranges in this area. The glacier ranges from 1979 depicted on photographs, the glacier ranges in 2007 surveyed using Global Positioning System (GPS) techniques and ranges from 1956 based on aerial photographs were also shown on this map.

Changes in glaciers ranges on King George Island, encompassing glaciers in Admiralty Bay, are presented in the studies by Braun [13] and Rosa [14]. The subject matter of contemporary glaciers changes beyond Admiralty Bay or the whole of King George Island was researched in these studies [15,16,17,18]. Over previous decades, [19,20] have described climate change on King George Island. The influence of climate change on glaciers geometry in King George Island was presented in [21,22,23,24].

This paper presents the results of our studies on changes in the range and thickness of glaciers in the Western shore of Admiralty Bay over the last few decades. Archival aerial images and satellite imagery from the Pleiades system, as well as surveys performed using GNSS (Global Navigation Satellite System) techniques and terrestrial laser scanning, were used in this study. The main aim of this study is an analysis of the impact of the glacier morphology on the size and rate of observed changes. The paper contains considerations regarding the important role of the longitudinal slopes of the glaciers’ surfaces in the connection of changes in the thickness of the glaciers and changes in the location of their fronts. The work particularly refers to the results of glacier studies in the western shore of Admiralty Bay [25,26,27] published in recent years. Mentioned works present in detail the changes in the thickness and recession rate of the Ecology Glacier [25] and the Baranowski Glacier [26], respectively. These works concern individual glaciers without reference to others located in the vicinity, thus there is a lack of discussion about the impact of their morphology on the rate of observed changes. There was a chance for this kind of analysis in the third work [27], which already includes all glaciers in the western shore of Admiralty Bay, representing several morphological types of glaciers. However, this opportunity was missed because there are no conclusions drawn about the impact of morphological features on the recession rate of various glaciers. The authors did not study the rate of glacial recession, but studied the rate of loss of glacial surface for different morphological types of glaciers. Pudełko [27] did not study changes in glacier thickness and the impact of morphology on these changes. All of the above-mentioned missing analyses have been performed in our work and therefore, our research and these existing works complement each other, providing full knowledge about the studied glaciers.

The motivation of our paper is a comprehensive overview at changes of few glaciers, and not a single as in cited works. The selection of the studied glaciers is not accidental, as they are representative in terms of the type of front and longitudinal slope. This allows for the formulation of new and interesting conclusions regarding the impact of glacier morphology on the rate of changes in glacier ranges and their thickness, and other parameters of these changes. The most interesting value of this work is the recognition of the role of glacier slope in the connection of glacier thickness changes and their recession.

## 2. Research Area

### 2.1. Geographical Characteristics

King George Island’s ice cover consists of several connected ice caps with pronounced and numerous outlet glaciers. King George Island is the largest of the South Shetland Islands, with a surface area of about 1310 km^2^, 90% of which is permanently glaciated.

**Figure 2:**
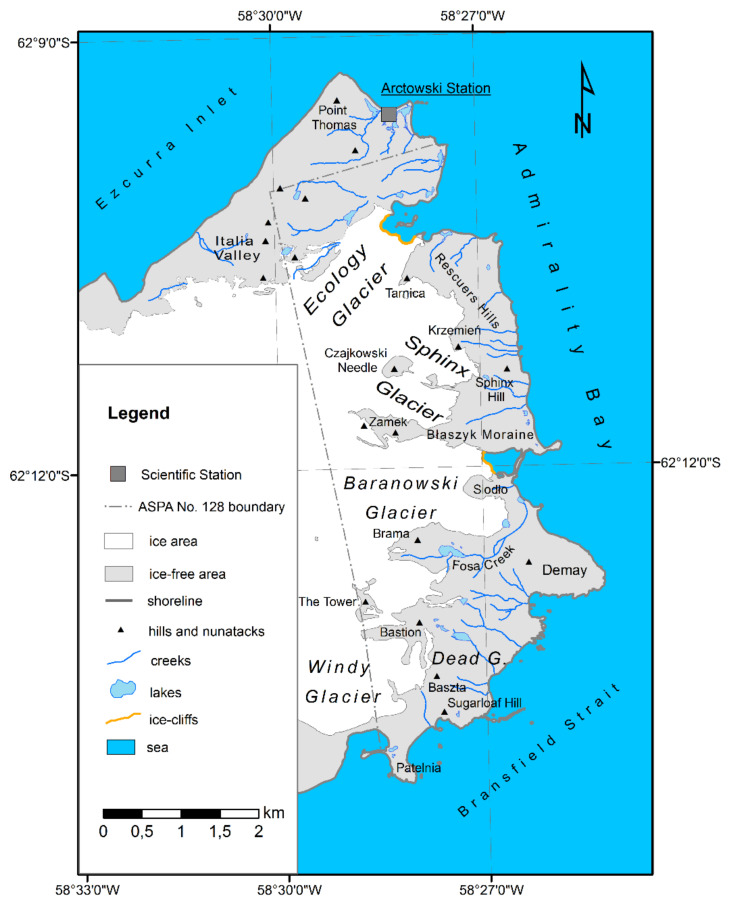
Antarctic Specially Protected Area (ASPA) No. 128 and glaciers in this area.

**Figure 3:**
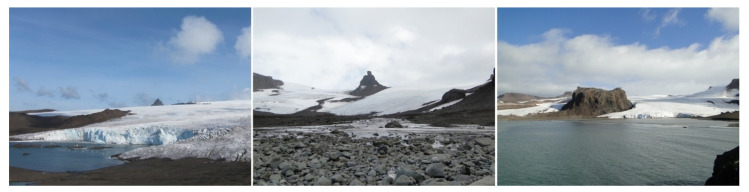
Ecology Glacier (**left**), Sphinx Glacier (**middle**) and the Baranowski Glacier (**right**) (photos taken by M. Kejna, L. Urbański, M. Kowalska) in 2015.

Six glaciers fed by the Warszawa Icefield (reaching approx. 500 m a.s.l.) are present in ASPA No. 128 (Figure 2) [28]. The ice cover in the central part of the west border of the analyzed area reaches a height of 300–350 m a.s.l. Two major glacier types can be distinguished in this region: land-terminating and tidewater glaciers [13,21]. Two ice streams originate from the icefield, which create outlet glaciers: the mostly grounded tidewater and partly land-terminating Ecology Glacier and the currently fully land-terminating the Baranowski Glacier (Figure 3). They are terminated by high calving ice cliffs (40–45 m and approximately 20 m high, respectively). The classification of the Ecology Glacier presented by us refers to the present state and is somewhat divergent with the classification of this glacier in other works. Braun and Grossmann [13] classify this glacier as land-terminating, however, large parts of its front terminate into the vast lagoon.

Two of the glaciers mentioned above are divided by a vast ice patch, which constitutes the marginal part of the icefield. This patch is limited from the north by Rescuers Hills, from the west by Krzemień and Sphinx Hill, and in the southern part it slopes down gently to the ground as the front of Sphinx Glacier (Figure 3). Sphinx Glacier is separated from the Warszawa Icefield by two nunataks: Czajkowski Needle (also known as Pawson Peek) and Zamek (also known as Anvil Crag). A medial moraine known as Błaszyk Moraine stretches towards the shore behind Zamek nunatak, dividing Sphinx Glacier from the Baranowski Glacier. Siodło nunatak divides the Baranowski Glacier into two parts. The northern part is characterized by a cliff front, whereas the southern part slopes down towards Fosa Creek. The glaciers located in the southern part of ASPA No. 128, i.e., Tower, Dead and Windy, are not subjects of this study.

### 2.2. Climate Changes

King George Island is characterized by some of the greatest temporal variations in climate conditions in the Southern Hemisphere [20,29,30]. The island is characterized as a polar climate (tundra climate type ET according to W. Köppen’s classification), with a strong marine influence. The average air temperature at the Bellingshausen Station located on the Southern Shore of King George Island in the period 1968–2016 was 2.5 °C (data from Arctic and Antarctic Research Institute, St. Petersburg, Russia). The summer on the island is short; February is the warmest month (average 1.5 °C) and July is the coldest (average −6.4 °C) at this station. There are high levels of precipitation; in the same period, the average yearly value was 697.9 mm (Bellingshausen Station). Climate conditions at the western shore of the Antarctic Peninsula are characterized by significant variability connected to the sea ice ranges and atmospheric circulation [20].

**Figure 4:**
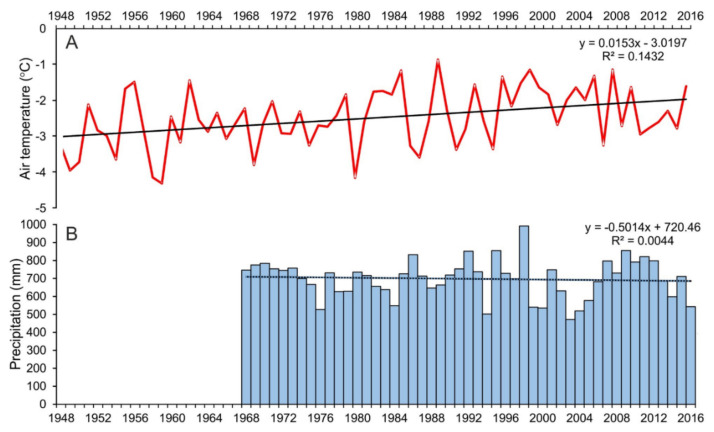
Charts present: (**A**) course of mean annual air temperature on King George Island (1948–2016), (**B**) annual precipitation at the Bellingshausen Station (1968–2016).

The fastest increase in air temperature in the whole of the Antarctic can be observed in the Antarctic Peninsula region. One of the largest air temperature trends of 0.54 °C/decade during the period 1951–2011 was recorded at Faraday/Vernadsky station [31]. Based on a joint series from the stations operating on King George Island (methodology described in the article by Kejna [20]), it has been found that in the period between 1948 and 2016, the mean annual air temperature increased by as much as 1.0 °C (trend 0.15 °C/decade). The period from 2001 to 2010 was the warmest decade (average −2.0 °C). The warming is characterized by a higher frequency of days with an air temperature above 0 °C, which causes longer ablation periods. A slight cool down (average −2.5 °C in the period 2011–2016) in recent years can be observed (Table 1, Figure 4). In the period 1968-2016, annual precipitation in this region does not show any significant trend (Table 1, Figure 4). 

Heavy thaws occurred frequently during winter and caused melting of the snow cover, especially in the lower parts of the glaciers. This influences snow accumulation and total glacier mass balance. During colder summers (e.g., 2012–2013), contrary to the dominating trends of the increase of the air temperature, surface mass balance remains positive [21]. The atmospheric parameters mentioned above and additionally positive degree days (PPD) registered at the other station on King George Island in the period 1968–2014 were presented by Pudełko [27].

## 3. Materials and Methods

The parameters which define changes in the geometry of the tested glaciers (changes in ranges and changes in the glacier thickness) are determined in this work. Changes in ranges and the thickness of glaciers observed over the time periods allowed our research to also determine the average rate of these changes in these intervals. The changes in glacier thickness were determined from the differences of Digital Surface Models (DSMs), presenting the landform containing glaciated areas. Although DSM is the proper name for the elevation model used here, Digital Elevation Model (DEM) is the term used later in this work, because this is the most common term used in glaciological literature for bare ground and glacier surface descriptions.

### 3.1. Source Data and Processing Methods

**Figure 5:**
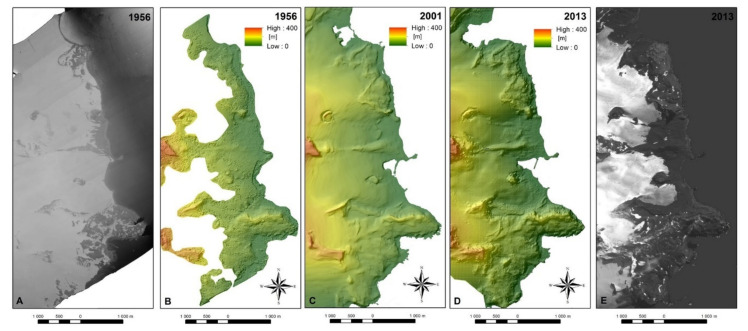
Visualizations of the selected studies used in the analysis: (**A**) orthophotomap and (**B**) digital elevation model (DEM) obtained using Falkland Island Dependency Aerial Survey Expedition (FIDASE) aerial photography from 1956; (**C**) DEM from 2001 created based on the map at a scale of 1:12,500 [10]; (**D**) DEM and (**E**) orthophotomap generated using images from the Pleiades-1A system collected in 2013.

To carry out analyses of changes in glacier geometry at ASPA No. 128, cartographic, surveying, airborne and satellite data were used (Table 2). This data was used to determine the glaciers’ ranges and changes in their thickness. Aerial photographs taken on 20th December 1956 by the British Government Ministry of Overseas Development, as a part of the Falkland Island Dependency Aerial Survey Expedition (FIDASE), were the oldest set of data used in this study. Based on these photos, a point cloud characterized by densities 1–2.5 points per 10 square meters and an orthophotomap (Figure 5A) were generated using Agisoft Photoscan software with pre-processing of analogue images using the SAPC application [32]. The DEM was generated from point clouds using ArcGIS software (Figure 5B).

The application of automatic image matching algorithms, which do not cope well with ice surfaces characterized by the uniform texture of their images, resulted in the DEM covering only a several hundred-meter-wide area along the western shore of Admiralty Bay. The model visualized only the frontal fragments of Ecology and the Baranowski Glaciers. Nevertheless, it was possible to perform analyses on the thickness changes in this case.

An orthophotomap at a scale of 1:10,000 [11,12] based on aerial photographs taken on 9 February 1979 by the Ecology Institute of the Polish Academy of Sciences using BAF and AFA cameras, was also used. The mentioned photographs were used to illustrate the glaciers’ ranges in the summer of 1978/1979. The map also contains ranges of the glaciers from 1956, obtained from FIDASE photographs, which have already been presented.

GPS surveys performed using the RTK (Real Time Kinematic) technique carried out by Pudełko, and tacheometric measurements from the summer of 2000/2001, as well as a topographic map at a scale of 1:12,500 for SSI-8 (currently ASPA No. 128) created based on these, served as another source of data [10]. GPS and tacheometric measurements were used to depict glacier ranges, whereas the digitalized landform presented on a topographic map was used by Pudełko to create a DEM using ArcGIS software (Figure 5C) which was applied in three-dimensional analyses. Measurements carried out in 2007 using the same techniques [12] were also used in analyses of the changes in the glaciers’ ranges.

Analyses of glacier ranges in the last decade were based on single high-resolution satellite images taken on 2nd February 2011 and 21 January 2014 by GeoEye-1 system, as well as on images taken by Pleiades-1A on 13th March 2013. As the Pleiades system provided triplet images. They were used to generate the DEM (Figure 5D) for analyses of changes in ice thickness and the orthophotomap (Figure 5E) for analyses of changes in the ranges. Both products were created in ERDAS IMAGINE Photogrammetry software.

The range of the applied methods was supplemented with terrestrial laser scanning (TLS) carried out using the Leica Nova MS50 total station which was used for the Ecology Glacier range imaging in March 2015. The GNSS RTK technique was used to register the ranges of the remaining glaciers in this study. In order to increase the representability and accuracy of the research results, data presenting the condition of the glaciated areas during the summer or towards its end was used. Thus, the data approximately characterized the condition of the glaciers at the end of the hydrological year.

### 3.2. Data Accuracy

Accuracy of the source data is dependent on the measuring technique which was applied. In the case of range analyses, the accuracy of points determining the border of glaciated areas was investigated. Where ice surface elevation changes are concerned, the DEMs’ accuracy was investigated. In the theoretical analysis, estimated values of the individual elements determining the final accuracy of the ranges or DEMs were used.

Aerial photographs were the oldest type of data used for analyses of range changes. Horizontal accuracy of orthophotomaps created based on those images depends mainly on their spatial resolution, which results from the scale of the photograph related to the altitude at which they were taken, and scanning resolution, as well as surveying precision of the ground control points (GCPs) coordinates used to transform the study to a global reference system. The photographs from 1956 were taken at a scale of 1:27,000. They were made available in a scanned (digital) form with a resolution of 25 µm (1000 dpi), which provides a ground sampling distance (GSD) of approximately 70 cm. Information on the scale and resolution for the photographs from 1978 to 1979 is not available, however, similar parameters for the digital images as above should be expected.

Both the photographs from 1956 and 1979 were studied with the use of GCPs measured using GPS or tacheometry. The horizontal control point measurements of these techniques are accurate to a few decimetres. Additionally, taking into consideration the errors resulting from the photographs’ resolutions, and the fact that ranges are a discretization of their continuous image reflected in the photographs, it can be assumed that their horizontal accuracy is within 2–3 m. Estimated accuracy for the photographs from 1979 by Pudełko [27] is 2 m.

A slightly better horizontal accuracy characterizes the ranges obtained using GPS or tacheometric measurements in 2001, 2007 and 2015. Image resolution inaccuracies are avoided when using these methods, thus the precision of the ranges from those time periods presented in this paper is 2 m. Similar accuracy (1–2 m) has been given for these measurements by Pudełko [27]. Improved horizontal accuracy characterizes the glacier ranges determined using terrestrial laser scanning in 2015. Despite the centimeter accuracy of this technique in polar conditions, spatial orientation is based on GPS and tacheometric observations, thus resulting in an accuracy of 1 m.

The horizontal accuracy of glacier ranges determined using satellite images from 2011, 2013 and 2014 depends on their spatial resolution and the accuracy of geometrical corrections and orthorectification processes, as well as on the GCPs’ accuracy. Considering the deterioration of accuracy of the ranges resulting from discretization, one can expect the accuracy of the determined ranges to match that obtained for aerial photographs, which is within 2–3 m. Pudełko [27] estimated accuracy glacier extents using satellite images at 2 m.

Elevation accuracy in the DEMs is estimated at approximately 3 m for aerial photographs from 1956, 5 m for the topographic map from 2000/2001, and at 1 m for satellite images from the Pleiades system obtained in 2013. Fieber [33] estimated the DEM’s accuracy at 1.82 m for the WorldView-2 system and 2.57 m for FIDASE imagery. Pętlicki [25] calculated similar empirical errors for the DEM based on satellite images from the Pleiades system from 2012 (1.27 m) and the topographic map from 2001 (6.61 m), and a slightly larger error for the model obtained from aerial photographs taken in 1979 (9.70 m). Similar values of DEM error based on Pleiades images (1–1.5 m) were reported in Postelniak [34] and Sofia [35]. A review of the work on DEMs’ accuracies based on multisource data and the use of them to study changes in glacial geometry can be found in Bolch [36].

The DEM vertical accuracy was empirically controlled in this study using a test field located near Arctowski Antarctic Station. In 2015, in the surroundings of the station, a DEM was created using data from TLS with a resolution of 0.2 m and accuracy of 0.05 m in comparison to control measurements using the GNSS RTK technique and tacheometric surveys [37]. As the area is devoid of ice cover and there is no evidence of any significant changes in terrain over the last 50 years, it serves as a good test field for other models. For the 2001 and 2013 models, the maximal obtained height discrepancy was approximately 2 m. This confirms the accuracies of these models, previously described as theoretical considerations.

No empirical accuracy verification was performed for the DEM from 1956 because the area of the studied aerial photographs did not cover the test field. It should be emphasized that the test field is small and characterized by low terrain denivelations. It is slightly disadvantageous in the context of utilizing this area for DEM analysis, but it is the only area for which data enabling empirical verification of theoretical considerations concerning their accuracy is available.

### 3.3. Other New Methods

It can be noted that interesting and original parameters regarding the distribution of glacier thickness reductions, which have not yet been presented in other cited works, are two components (longitudinal and vertical) of the thickness reduction gradient. The decrease of the ice thickness reduction (*d*Δ*T*) in meters with increasing distance from the front (*dL*) in meters is the longitudinal thickness reduction gradient. In turn, the decrease of the ice thickness reduction (*d*Δ*T*) in meters with increasing height of the glacier surface (*dH*) in meters is the vertical thickness reduction gradient.

The relationship of these two components of the thickness reduction gradient were obtained in accordance with Formula (1):

(1) $$\frac{d\Delta T\;}{dH}=\frac{d\Delta T\;}{dL}\frac{dL}{dH}=\frac{d\Delta T\;}{dL}\frac{1}{S}$$

where *d*Δ*T* is the value of the glacier thickness changes, *d*Δ*T/dH* is the vertical thickness reduction gradient, *d*Δ*T/dL* is the longitudinal thickness reduction gradient, and S is the slope of the glacier surface.

Most often, changes in atmospheric conditions have been indicated as the main cause of visible changes in the glaciated area in the form of glacier front recession and their thickness changes. Among the factors that differentiate the influence of atmospheric changes, glacier morphology and their thermal structures were listed. In turn, an important factor impacting purely geometrically on the rate of front recession is the longitudinal slope of the glacier surface in the frontal zone. It is a kind of connector between the rate of ice thickness changes in that zone and the rate of the changes in the glacier front position in accordance with Formula (2):

(2) $$\frac{dL}{dt}=\frac{dH}{dt}\frac{dL}{dH}=\frac{dH}{dt}\frac{1}{S}$$

where *dL/dt* is the velocity of displacement of the glacier front in the longitudinal direction, *dH/dt* is the velocity of change in glacier surface elevation and *S* is the glacier longitudinal slope in the frontal zone.

Velocities *dL/dt* is the same velocities which Venkatesh [38] presented as the sum of the velocity caused by the gravity-driven ice-flow (dynamic effect) and the velocity caused by the influence of the mass balance (thermodynamic effect) which additionally resulting in changes of equilibrium line altitude (ELA). Similarly, velocity *dH/dt* is the result of both of these effects. Velocities *dL/dt* and d*H/dt* are in a specified period result in the observed size of the front recession (*Fr*) and the ice thickness reduction (Δ*T*). It follows that the size of the recession is determined by the ratio of the ice thickness reduction to its slope (3):

(3) $$Fr=\frac{\Delta T}{S}$$

Therefore, decreasing the slope causes an increase in the recession rate, with the same loss of ice thickness.

**Figure 6:**
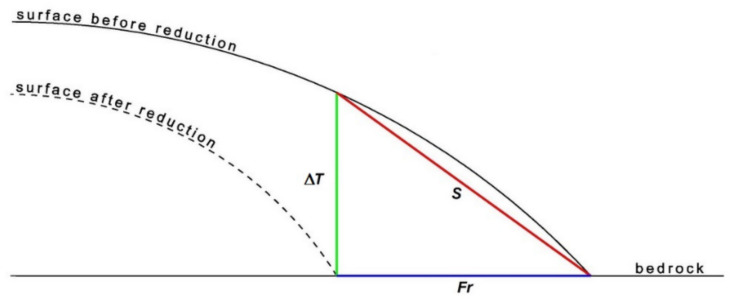
Relationship between the glacier thickness reduction (Δ*T*), glacier surface slope (*S*) and the size of the glacier front recession (*Fr*).

The essence of this relationship of glacier thickness reduction and its front recession is shown in Figure 6. The above relationships are identical in the case of the increase in the ice thickness and the front advance caused by it. Despite the varying levels of detail, both approaches—presented in this work and proposed in [38]—for analyzing impact of the slope on the range changes are closely connected.

## 4. Results

### 4.1. Changes in the Glaciers’ Ranges

**Figure 7:**
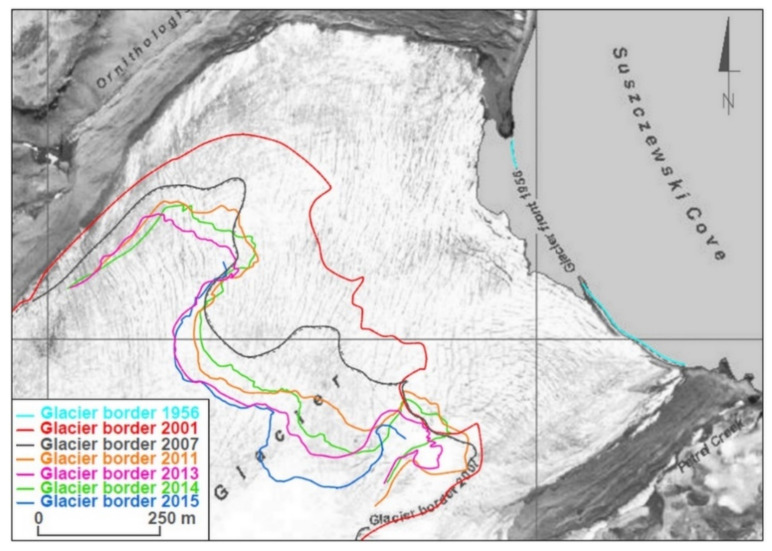
Changes in the Ecology Glacier’s front range over the period 1956–2015 on a background in the form of an orthophotomap created by Pudełko [11]. The glacier ranges shown on the photograph represents the ranges in 1979.

**Figure 8:**
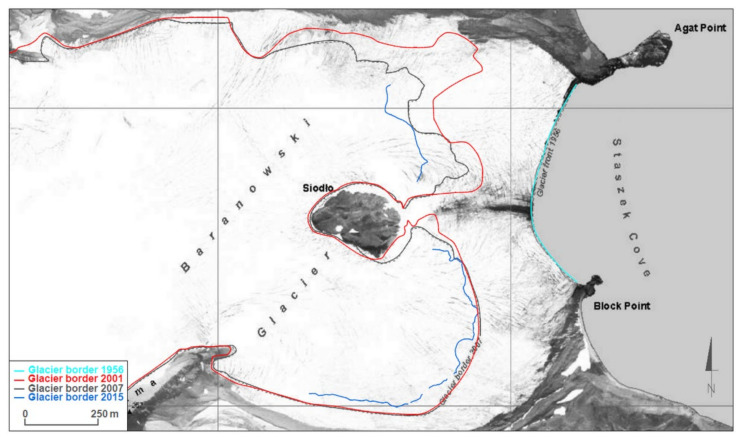
Changes in the Baranowski Glacier’s front range over the period 1956–2015 on a background in the form of an orthophotomap created by Pudełko [11]. The glacier ranges shown on the photograph represents the ranges in 1979.

**Figure 9:**
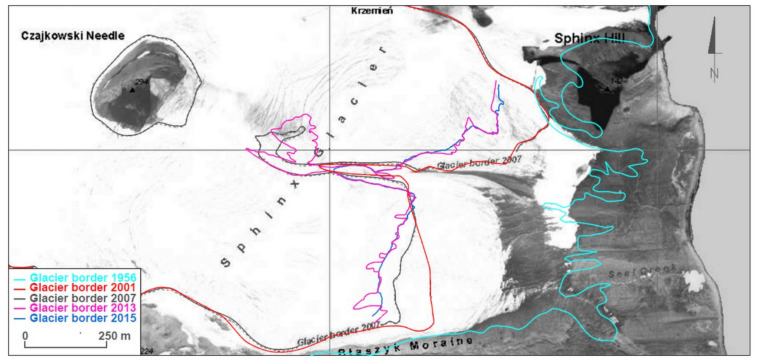
Changes in Sphinx Glacier’s front range over the period 1956–2015 on a background in the form of an orthophotomap created by Pudełko [11]. The glacier ranges shown on the photograph represents the ranges in 1979.

An orthophotomap at a scale of 1:10,000, produced by Pudełko, was chosen to serve as a starting point for analyses of glacier range changes [11,12]. This map shows glacier ranges in 1979, when the aerial photographs used to create them were taken. Additionally, glaciers ranges based on FIDASE aerial photographs from 1956 and ranges from 2007 measured utilizing GPS and tacheometric techniques were marked on the map. The image was completed with ranges from 2001, 2011, 2013, 2014 and 2015 (Figure 7, Figure 8 and Figure 9).

The rate of changes of the Ecology Glacier front is characterized by clearly visible variability over time (Figure 7). The retreat in the initial stage of the analyzed period is significantly slower. In the period 1956–1979 the glacier’s front remained unchanged in the southern part of the cliff. However, in the northern part, the cliff receded by approximately 100 m. This amounts to an average recession rate in this area of 5 m a^−1^. Between 1979 and 2001 the glacier’s front—this time over its full width—retreated by another 350–400 m, with the average rate increased to approximately 20 m a^−1^.

The period 2001–2015 was also characterized by irregular recession of the glacier’s front. While the glacier’s tongue is land-terminated, the retreat was at average rates of 15 m a^−1^ (on the northern side) and 5 m a^−1^ (on the southern side). The highest recession took place in the central part of the front, where the ice-cliff terminates to a lagoon filled with meltwater and sea water from Suszczewski Cove. In this period, the highest spatial difference of the retreat rate was observed for the central zone of the glacier’s front. The northern part of the central zone of the Ecology Glacier front retreated at an average rate of 40 m a^−1^ until 2007, then the recession rate decreased to approximately 15 m a^−1^. In comparison, in the southern part of the central zone, the average retreat rate of the front—over the same period—was almost reversed. Between 2014 and 2015, a record annual advance of the front of 120 m was observed. Overall, in the period 2001–2015, the Ecology Glacier retreated by another 250–400 m, which is an average recession rate of 15-25 m a^−1^. Nevertheless, this period also witnessed a significant advance on the whole width of the front between 2013 and 2014, which was approximately 40 m (reaching 70 m in the northern part of the central zone of the ice-cliff).

At present the northern part of the Baranowski Glacier’s tongue which descends into Staszek Cove in the form of the land-terminating ice-cliff is divided by the Siodło nunatak from the southern part, which terminates gently onto the land. These differences between the northern and southern parts of the glacier are reflected in the retreat dynamics of the front of this glacier (Figure 8).

From 1956 to 1979, the range of the Baranowski Glacier’s marine-terminating cliff remained unchanged along its whole length. Then until 2001, the glacier’s front receded by 200–400 m, both in the northern and southern part. Therefore, the average recession rate in that period was 10–20 m a^−1^. From 2001, a noticeable change in the dynamics of each part of the glacier was observed. Until 2007, the southern part showed no changes in its range. Then until 2015, a minor retreat not exceeding 100 m was registered with an average change rate of approximately 5 m a^−1^. In contrast, from 2001, the northern part retained its average recession rate of 10–20 m a^−1^, retreating by another 100–300 m. Observed differences in the behavior of both parts of this glacier are probably caused by the fact that currently only the northern part has an ice-cliff front, which additionally, already ends on the land. According to Sziło and Bialik [26] the southern part became land-terminating around 1980.

A decrease in the width of the Ecology Glacier’s tongue by 200–300 m (which had an original width of approximately 1 km) was observed during the period 1956–2015. In comparison to the Ecology Glacier, the reduction in the width of the Baranowski Glacier’s tongue was slightly smaller: 100–200 m. Its original width was 1200–1300 m.

The whole front of Sphinx Glacier ends on land, which is a reason why it is characterized by different dynamics to the two earlier glaciers (Figure 9). The range for Sphinx Glacier in 1956 was not marked on the orthophotomap created by Pudełko [11], so the information was filled in based on the obtained British aerial photographs. In the period 1956–1979, the glacier front receded by 100–200 m; an average rate of 5–10 m a^−1^. In the period 1979–2001, the glacier retreated by another 200–250 m; an average rate of about 10 m a^−1^. In the following years, the process slowed down, and between 2007 and 2013 the front receded by 50–100 m; an average rate of 10–20 m a^−1^. In the years 2013–2015, once again, no significant changes in the location of the Sphinx Glacier’s front were observed.

### 4.2. Changes in the Glaciers’ Thicknesses

**Figure 10:**
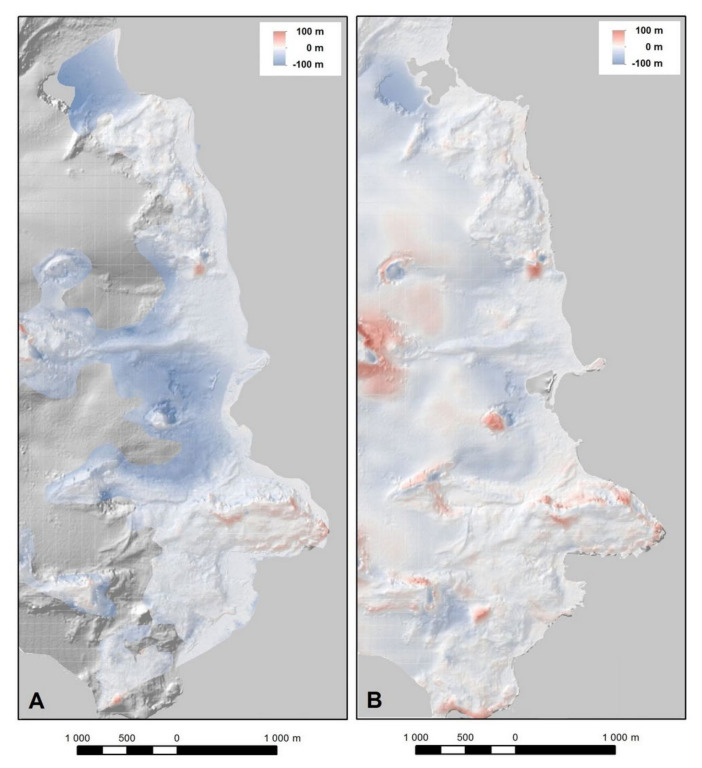
Differences between digital elevation models (DEMs) (**A**) for 1956 and 2013, (**B**) for 2001 and 2013. Negative values denote loss of ice thickness, positive values denote an increase.

Changes in the glaciers’ thicknesses were analyzed using the available DEMs for 1956, 2001 and 2013. We analyzed the DEMs’ differences in the periods 1956–2013 (Figure 10A) and 2001–2013 (Figure 10B). The selection of the first period of analysis stemmed from the desire to show the scale of the reduction in glacier elevation during the last 50 years. Due to the spatial limitations of the DEM from 1956, the analysis of changes for this period mainly shows the glaciers’ surface elevations where no ice is present. The second period was chosen because of the short time difference of data acquisition, which presented current dynamics of thickness changes. We decided not to present changes in subsequent time periods covering the entire period of 1956–2001, because it is such a long period that the potential conclusions concerning the rate of changes would be highly averaged and would not reflect the real rate of the changes.

Differences between the DEMs for 1956 and 2013 illustrated that in ASPA No. 128, in places where the 1956 model was generated, the highest changes in the thickness of the glaciated areas took place in the region of Ecology and the Baranowski Glaciers (Figure 10A). A significant reduction of the ice surface elevation within those glaciers can also be observed when analyzing the differences between the DEMs from 2001 and 2013 (Figure 10B). In addition, for these periods, large changes are visible in the Sphinx Glacier.

**Figure 11:**
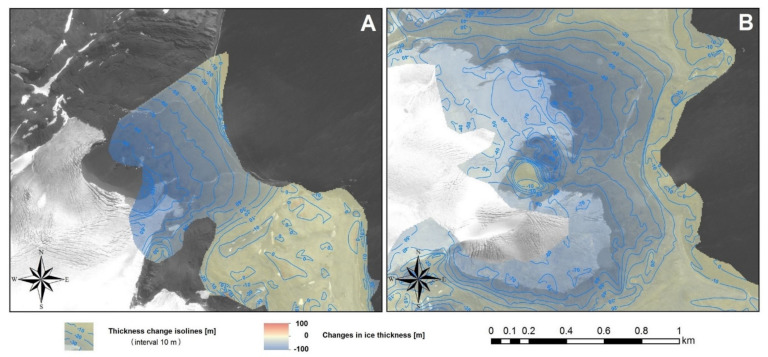
Differences between digital elevation models (DEMs) for 1956 and 2013: (**A**) in Ecology Glacier and (**B**) in the Baranowski Glacier presented in color scale with isolines. Negative values denote loss of the glacial thickness, positive values denote an increase. The panchromatic orthophotomap obtained based on satellite images from Pleiades system recorded in 2013 was added as a background, for presentation of an approximate current position of the glaciers’ fronts.

The results of detailed analyses of the areas of the largest differences of the DEMs for 1956 and 2013 are shown in Figure 11. Figure 11A presents elevation changes for the Ecology Glacier in places where the ice is no longer present. Therefore, these figures represent the elevation of this glacier from 1956 above sea level. At that time, the glacier’s cliff reached 30–40 m a.s.l. Differences between the DEMs are presented in a color scale with isolines. A panchromatic orthophotomap obtained from satellite images taken in 2013 was used as the background for these figures. This allowed for an estimation of where the greatest changes could be located. This glacier was approximately 120 m a.s.l. in 1956. Currently, the cliff measures 40–45 m a.s.l.; therefore, the loss of ice thickness is 75–80 m. Slightly lower losses were observed in the Baranowski Glacier region (Figure 11B). In 1956, the cliff of this glacier (the northern part) reached about 30–40 m a.s.l. As with the Ecology Glacier, in the northern part of the Baranowski Glacier the highest changes occurred near the glacier front in 2013. In the place where the glacier front was in 2013 it was approximately 80–90 m a.s.l in 1956. The current height of the cliff is equal to 15–20 m a.s.l, so the loss in the ice thickness is 60–75 m. The same changes were observed in the southern part of this glacier.

**Figure 12:**
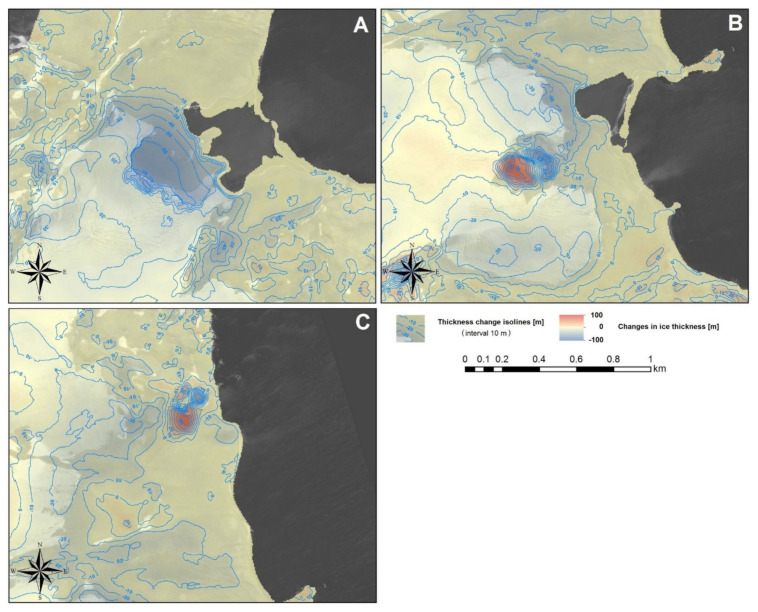
Differences between digital elevation models (DEMs) for 2001 and 2013: (**A**) around the Ecology Glacier, (**B**) around the Baranowski Glacier and (**C**) around Sphinx Glacier presented in color scale with isolines. Negative values denote loss of the glacier’s thickness, positive values denote an increase. The panchromatic orthophotomap obtained based on satellite images from the Pleiades system recorded in 2013 was added as a background, for presentation of an approximate current position of the glaciers’ fronts.

Based on the differences between the DEMs for 2001 and 2013 (Figure 12), it has been found that in 2001 the Ecology Glacier’s cliff reached 30–40 m a.s.l., whereas the Baranowski Glacier’s cliff was approximately 20–30 m a.s.l. In the period 2001–2013, the reduction of the ice thickness occurred only in the frontal zone of the glaciers. For glaciers with an ice-cliff front (Ecology and the northern part of Baranowski), the largest reduction in ice thickness (about 70 m and 40 m, respectively) was observed below the range line from 2013. The reduction was not related only to surface ablation, but also to the front retreat caused by calving and the thermo-abrasive influence of water. Hence, data for these glaciers from this zone cannot be a good indicator of the rate of change in glacier thickness caused by ablation on their surface because of climate changes. They are rather an indicator of the glacier volume loss caused by the glacier front retreat. The largest reduction of the ice thickness for land-terminating glaciers (Sphinx and the southern part of Baranowski) was observed above the range line from 2013. This is because the thickness changes above this border (in the current frontal zone) were the main results of the climatic impact on glacier thickness changes in this paper, regardless of the front type. In this zone, the maximum thickness reduction was 20–30 m in the case of the Ecology Glacier (Figure 12A) and the northern part of the Baranowski Glacier, and 10–30 m for the southern part of the Baranowski Glacier (Figure 12B) and Sphinx Glacier (Figure 12C). In the upper parts of the frontal zone, the changes were lower.

### 4.3. Other Characteristics of the Glacier Thickness Changes

The approximate average values of these gradients above the glaciers’ fronts in 2013 (calculated in the direction of the glacier top according to the Formula (1)) are presented in Table 3.

**Figure 13:**
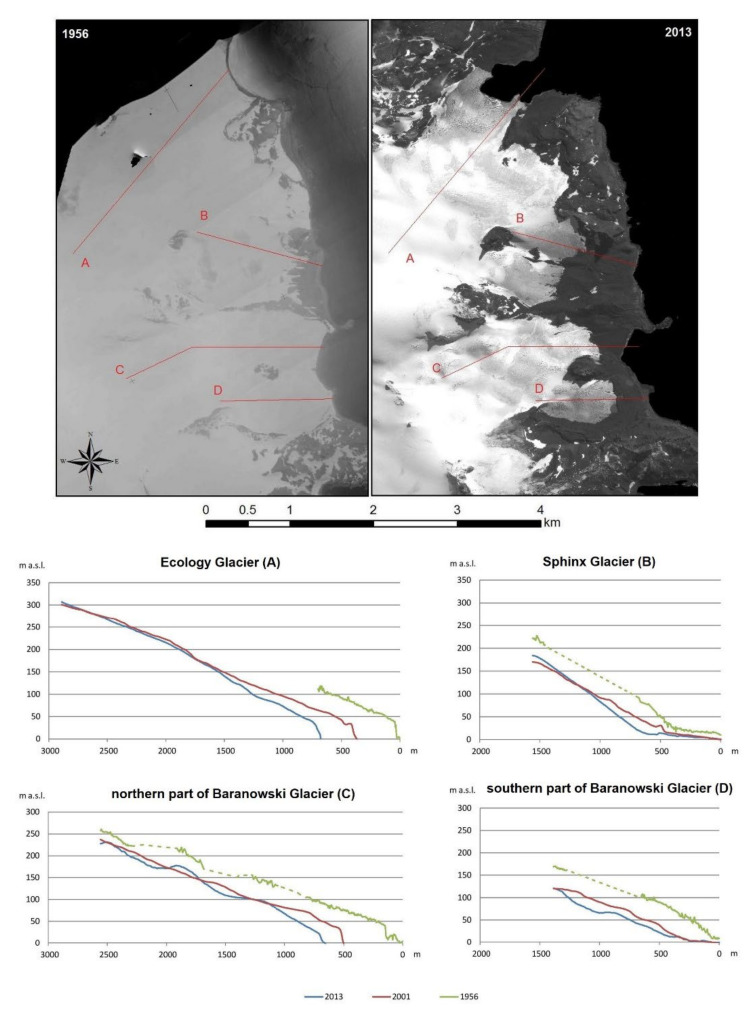
Longitudinal elevation profiles based on digital elevation models (DEMs) from 1956, 2001 and 2013: (**A**) for Ecology Glacier, (**B**) for Sphinx Glacier, (**C**) for northern parts of the Baranowski Glacier and (**D**) for southern parts of the Baranowski Glacier with their location. Profile line placements are presented on the orthophotomap based on Falkland Island Dependency Aerial Survey Expedition (FIDASE) aerial photographs from 1956 and the orthophotomap from the Pleiades system registered in 2013 (the starting point for profiles on the frontal side in 1956). The dashed line shows the course of interpolated profiles between the known fragments of the DEM for 1956.

They were estimated based on the maximum thickness reduction in frontal zone for all glaciers in the period 2001–2013 (about 30 m), the average width of their thickness reduction zones (ablation zones) for the period 2001–2013 and approximate values of the glaciers’ surface slope (in 2013 calculated along longitudinal profiles presented in Figure 13). Because the maximum thickness reductions in the frontal zone above range line from 2013 were used to determine the average gradients, therefore the widths of reduction zone were measured from this line for these calculations. The widths of the reduction zone were determined on the basis of Figure 12 and Figure 13.

Both longitudinal and vertical thickness reduction gradients were clearly higher for both parts of the Baranowski Glacier and Sphinx Glacier (Figure 12B,C and Figure 13B–D) than for the Ecology Glacier (Figure 12A and Figure 13A).

It is clearly visible in the longitudinal elevation profiles of these glaciers based on the DEMs for 1956, 2001 and 2013 that significant changes in glacier thickness took place over their whole length in the period 1956–2001, but only in the glacier frontal zone in the period 2001–2013 (Figure 13). In the first period, the reduction in ice thickness over the entire length of the profiles reached a similar value of about 40–50 m. In the second period, the reduction reached a maximum of approximately 30 m, as already described in detail in the analysis of the differences of the elevation models. For higher parts of the glaciers, the differences are insignificant and within model error margins, which is considered in the discussion section.

In the period 2001–2013, only in the case of the Ecology Glacier can it be clearly concluded that the reduction in ice thickness took place to the altitude of approximately 170 m a.s.l. (Figure 13A). As for the other two glaciers, it is impossible to determine with certainty based on elevation profiles the height to which the ice thickness is reduced. For Sphinx Glacier, reduction occurs up to about 120 m a.s.l. (Figure 13B), just like for the southern part of the Baranowski Glacier (Figure 13D). This boundary in the northern part of the Baranowski Glacier is located at about 100 m a.s.l. (Figure 13C). Within these two glaciers, this border can be roughly determined by a line extending from the eastern end of Brama nunatak to the north in the direction of Czajkowski Needle, then in the north-eastern direction towards Krzemień Hill.

The given ice thickness reduction values between 2001 and 2013 indicated that glaciers thickness above the front in 2013 decreased at an average rate of 1.7–2.5 m a^−1^ (for Ecology Glacier and the northern part of the Baranowski Glacier) and 0.8–2.5 m a^−1^ (for the southern part of the Baranowski Glacier and Sphinx Glacier).

### 4.4. Impact of Glacier Surface Slope and Ice Thickness Reduction on the Range Changes

Values of ice surface slopes in 2013 (neglecting the low differences in slopes between 2001 and 2013) were used as the basis for the approximate analysis of the slope and ice thickness reduction effect on the size of the front recession for the period 2001–2013. Since the longitudinal profile for Sphinx Glacier (Figure 13B) is not perpendicular to the contour lines describing the surface of the glacier and the varied slope in the northern and southern parts, the approximate slopes for both parts were determined based on a map by Pudełko [10]. The calculations for all of the glaciers’ recession size s, taking into consideration the impact of the slope and ice thickness reduction, were very close to the observed scale of the front recession of these glaciers for this period (Table 4).

The slightly greater recession observed for the Ecology Glacier, compared with that calculated based on the slope and thickness reduction, may be due to the significant impact of the calving and melting processes on the front recession, resulting from its contact with water. The difference between the calculated and observed values of the recession for this glacier shows the contribution of this impact reaching 30–40%. As can be seen from the southern part of the Baranowski Glacier and Sphinx Glacier, the values determined by the slope are slightly overestimated. However, it should be noted that, as the recession progresses, the slope in the frontal area rises, which was not included in the calculations. It should be emphasized that the level of coincidence between the calculated recessions (based on the thickness losses and glacier surface slope in the frontal area) and the observed size of the recessions can be considered as confirmation of the above thesis connecting these issues.

## 5. Discussion

### 5.1. Changes in the Geometry of Glaciers

The analysis of changes in glacier geometry in ASPA No. 128 indicates a significant recession in their ranges as well as a decrease in their thicknesses. In that area, there was clearly an increasing rate of retreat of glacier fronts, although this rate varied over time. The rate of retreat is also visibly dependent on the morphology of the glaciers. From 1979, the dynamics of the northern part of the Baranowski Glacier (with an average recession rate of 10–20 m a^−1^) resemble that of the Ecology Glacier (with an average recession rate of 15–25 m a^−1^) with one difference. Similarity in the front dynamics of both glaciers result from the fact that both glaciers (Ecology Glacier nearly on all its width and the Baranowski Glacier in its northern part) possess intensely calving ice-cliff fronts. The low difference in the strength of the front dynamics probably stems from the fact that Ecology is a tidewater glacier ending in the sea, whereas the Baranowski Glacier is currently a land-terminating glacier (however, in the past it had a marine-terminating front in its northern part similar to the one of the Baranowski Glacier). It seems that contact with water in the case of the Ecology Glacier causes faster calving due to thermo-abrasive influence of the lagoon waters, created by meltwater and sea water from Suszczewski Cove. The significance of this impact on the front recession may be confirmed by the correlation between maximum lagoon depth and the highest annual retreat rate in the southern part of the glacier’s frontal zone. Pętlicki et al. [25] speculated that the occurrence of the differences in depth may be due to the subglacial outflows of the fresh waters. These may additionally increase calving. Comparable values for the average retreat rate of the Ecology Glacier (18.8 m a^−1^ for the period 1979–2001 and 23.9 m a^−1^ for the period 2001–2012) were obtained by Fristad [24]. The front of Sphinx Glacier and the southern part of the Baranowski Glacier’s front were characterized by a gentle descent onto land and had a significantly lower rate of recession (5–10 m a^−1^) than the ice-cliff fronts.

Moreover, the reduction in ice thickness of those glaciers in the last period studied took place at a fast rate. Based on the analyses carried out as a part of this study, it was concluded that over the period 2001–2013 the mean annual rate of ice thinning in the frontal zone of Ecology and the northern part of the Baranowski Glaciers was 1.7–2.5 m a^−1^, and for the southern part of the Baranowski Glacier and Sphinx Glacier was 0.8–2.5 m a^−1^. A similar value (1.2–2.2 m w.e.) for the frontal zone of the Ecology Glacier for the period 2012–2013 was obtained by Sobota et al. [21] as a part of glaciological research of mass balance. Results of this study also state that the value of mass balance for the frontal zone of Sphinx Glacier was close to zero, which is reflected in the low changes in the range of this glacier in the period of 2013–2015 presented in this paper. Additionally, for the hydrological year 2012–2013, Sobota et al. [21] estimated the positive mean annual mass balance for Ecology and Sphinx Glaciers jointly, which was equal to +0.178 m w.e.

Similar values of reduction in ice surface elevation (18 m and 18.6 m) for the Ecology Glacier for the period of 2001–2012 were obtained, respectively, by Pętlicki [25] and Fristad [24], based on cartographic and satellite data. However, these values are mean values for the ablation area, hence are slightly lower than the maximum reduction values (20–30 m) given in this study for the comparable period of 2001–2013. It should be emphasized that the value and rate of glacier changes presented in this paper are consistent not only with the results of the latest research [24,25] but also with the results of other earlier studies. Kejna et al. [7] estimated that in the period 1978–1996, the recession of the Ecology Glacier was approximately 400 m, with a mean retreat rate of approximately 20 m a^−1^. The same rate was determined in this paper for the comparable period of 1979–2001. The study by Kejna et al. [7] also highlighted a reduction in ice thickness for the Ecology Glacier. Comparison of longitudinal profiles created using terrestrial techniques for 1978 and 1996 indicated a change in ice thickness in the frontal zone of the Ecology Glacier at 20–30 m, which gives an average rate of change of 2–3 m a^−1^ (a slightly lower rate of 0.8–2.5 m a^−1^ was estimated in this paper for the later period of 2001–2013).

The dynamics of changes for the studied glaciers are comparable to other glaciers on King George Island. Rosa et al. [15] estimated the recession rate for Wanda Glacier, a tidewater glacier that descends towards Martel Inlet in Admiralty Bay. The value of front retreat rate for this glacier was 22 m a^−1^, which is comparable to the recession rate described in this paper for the Ecology Glacier, which is characterized by an identical kind of front.

Interesting conclusions regarding the absolute height of the ice-cliffs of the Ecology and Baranowski Glaciers can be drawn from the analysis of the DEMs’ differences and longitudinal elevation profiles. While the values of thickness reduction in the current ablation zone for both glaciers are similar (both in the period 1956–2013 and 2001–2013), the changes which occurred in ice-cliff elevations were different. For the Ecology Glacier, the elevation of the cliff in both 1956 and 2001 was similar in the range of 30–40 m a.s.l., and in 2013 it even slightly increased to 40–45 m a.s.l. In the case of the northern part of the Baranowski Glacier, where it has a cliff, in 1956 it was 30–40 m a.s.l., in 2001 it decreased to 20–30 m a.s.l., and finally in 2013 it reached an elevation of 15–20 m a.s.l. It should be noted that the cliff elevations in 2001, mentioned here for these glaciers, are identical to those given by Batke et al. [22].

The significant differences between the DEMs in nunataks and the high decline areas and small positive differences in the ice-free areas of Demay Peninsula visible in Figure 10, Figure 11 and Figure 12 and are most probably caused by two reasons: imperfections in the model from 2001, which was created from the digitization of elevation data presented on a topographic map; and DEM errors for 1956 and 2013 based on aerial photographs and satellite images. In these areas, the information on the terrain in 2001 is incomplete (lack of contours) and was limited to the indication of the highest points. From other works (e.g., [25]) it is known that errors of the DEMs based on aerial images and satellite imagery in areas with large height differences can reach several tens of meters, caused by the crucial influence of horizontal error on vertical accuracy in sloped terrain. However, as the information for these areas does not concern the ice surface characterized by a gentle terrain relief, it is irrelevant to the presented analyses.

It should be added that, in the longitudinal elevation profiles for the Baranowski Glacier, above determined earlier border of the zone of the ice thickness reduction, low losses were noticeable both in Figure 12B and Figure 13C. There was also a slight increase in ice elevation for Sphinx Glacier above this border (Figure 13B). In both cases, slight losses and increases above this border should be largely attributed to errors in the differences of the DEMs due to previously mentioned model errors. It should be added that the fluctuation around the trend line of about 10 m, visible on the profiles for 1956, indicates a slightly worse DEM accuracy than is apparent from earlier theoretical considerations.

The ranges for Sphinx Glacier visible in Figure 9 are an excellent example confirming the high horizontal accuracy of the input data used in the performed analyses. This equally applies to the ranges registered directly in the field as well as those interpreted from aerial photographs and satellite images. High agreement between the ice ranges in periods characterized by marginal change dynamics deserves special attention. This concerns the periods 2001–2007 and 2013–2015. In the first case, the ranges in those years were determined by GPS RTK measurements supplemented by tacheometric surveys. For the second period, the ranges for 2013 were obtained from satellite images, whereas the ranges for 2015 were obtained from GNSS RTK measurements. The reported differences result from discretization of the ice ranges and difficulty in their identification in the field as well as in the registered images (aerial photographs and satellite imagery). The level of those differences is highly satisfactory regarding the size of the analyzed changes over the 1956–2015 period. One should also note the low range of elevation differences in the DEMs utilized in the study, oscillating around 0 in coastal areas, which were ice-free areas in 1956 (Figure 10). This serves as another confirmation of the high accuracy of the input data and of the analyses performed on their basis regarding the height dimension.

### 5.2. Differences in the Equilibrium Line Altitude (ELA)

The ELA obtained by Sobota et al. [21] for the Ecology Glacier equal 156 m a.s.l., determining its ablation zone for the 2012–2013 period, correlates with the results obtained in this paper. This result approximately determines the upper border for the zone of ice thickness reduction for this glacier, reaching around 170 m a.s.l. for the period 2001–2013. It should be emphasized that the determined zone of the ice thickness reduction does not refer to a single glacial cycle as the ablation zone limited by ELA, but to many cycles in the period 2001–2013. Therefore, the border of the glacier thinning zone is the average boundary, hence the low difference between the borders’ elevations. Foregoing compliance proves once more the complementarity of different analysis methods—both glaciological and geodetic. This conclusion is important because it indicates possible applications for remote geodetic techniques in the monitoring of glacial areas, where it is impossible to carry out glaciological terrestrial surveys, e.g., due to high risks.

The lower elevation (100–120 m a.s.l.) of the boundary delineating the zone of the ice thickness reduction was obtained for the Baranowski Glacier and Sphinx Glacier. Such significant difference, in the elevation of the thickness reduction zone border between these glaciers and the Ecology Glacier, correlates with large difference in the thickness reduction gradient of these glaciers. The lower value for both components of thickness reduction gradient for the Ecology Glacier (Table 3) means that the ice thickness reduction zone for the period 2001–2013 extends not only higher, but also further from its front (1000–1100 m) than in the case of the other two glaciers. For comparison, Pętlicki et al. [25] found that the thickness reduction zone for the Ecology Glacier extended to approximately 1500 m from the front for the period 2001–2012, and Sobota et al. [21] also found that width of the ablation zone for this glacier reached approximately 1000 m for the period 2012–2013.

The observed inverse correlation of the thickness reduction gradient value with the ELA value is very interesting. Based on this, it can be concluded that lower values of this gradient for a selected glacier compared to others are, like a high ELA value, an indicator of greater sensitivity of the glacier to the thermodynamic effect resulting from climate warming. This can be very useful because the comparison of ELA values requires determination of the net mass balance using glaciological methods for selected glaciers, which is not easy. On the other hand, components of the gradient can be compared using the differences of the DEMs or by performing longitudinal profiles, as it was shown in this paper.

Assuming that the obtained elevations of the upper border of the thickness reduction zone for the glaciers studied in this work are approximately consistent with the position of ELA for these glaciers, the results obtained for the Sphinx Glacier and Baranowski Glacier are very interesting. They suggest that the ELA for these glaciers is much lower than the lowest altitude (140 m a.s.l.) of this line of glaciers on King George Island presented by Pudełko et al.l. [27], and the ELA for the Baranowski Glacier (150 m a.s.l.) given by Sziło and Bialik [26].

### 5.3. Atmospheric and Geometric Influences

We think that glacier changes that were observed at some periods of time in this work are mainly the result of the thermodynamic effect, which is observed both as the front recession and thickness reduction—especially in terms of reduction in thickness because changes in the velocity of the gravitational flow of the glacier do not cause significant changes in thickness over a certain time period. The high compliance, between the observed fronts’ recessions and calculated as the slope and ice thickness reduction effect, confirms the conclusions from [38] that the slope is crucial in the response of small glaciers to climate change. However, it should be added that the slope affects not only the size of the response but also the time of the response. The total mass balance for glaciers also determines the size of the response. Both factors (slope and balance) interact with each other, suppressing or strengthening, depending on the mass balance, which can be positive or negative. Reducing the slope shortens the response time and reduces the advance due to the reduction in the dynamic effect arising from gravitational influence of the slope. The advance or retreat of the terminus due to the influence of the slope and the thermodynamic effect, which is dependent on the mass balance value. However, in the thermodynamic effect, the slope has an opposite impact than in the dynamic effect. Due to the variable direction of the thermodynamic effect, significantly higher advances can be expected as a result of the positive balance than recessions caused by the identical value, but with the negative balance. This may explain the observed advance of the Ecology Glacier between 2013 and 2014 which was several times greater than the average annual rate of recession for the period 2001–2015.

The advance of the Ecology Glacier front observed between 2013 and 2014 (Figure 7) was probably a response to the positive mean annual mass balance for the hydrological year 2012–2013 [21]. If this interpretation is correct, it would mean that the glacial response time is close to 1 year. Pelto and Hedlund [39] and Kulkarni et al. [40] indicated that the response time of other small glaciers varies between a few years and several decades. Prior to 2012, there were no mass balance studies, so it is certainly not possible to reject the possibility that this advance is a reaction to a slightly earlier positive mass balance, which would mean that the response time is slightly longer. If this time were longer than 1 year, then due to the positive balance for the period 2012–2013, the advance should be registered after 2014. In 2015 there was no frontal advance (Figure 7). No such advance was found in 2016 either, when analyzing the changes presented by Pętlicki et al. [25]. Thus, the response time would have to be 1 year or more than 3 years, which also cannot be excluded. The small size of the glacier (low length, width and thickness), slight slope of its surface, and a relatively large area of accumulation zone which increase the sensitivity of a glacier to climate change and shorten the response time [39,41], are favorable conditions supporting the suggestion of a 1 year response time. The Ecology Glacier meets all of these conditions, has a length of about 4 km [25], a width of about 1 km, a thickness in the frontal zone of about 40–45 m, a slope of about 0.1 in the frontal area, and an AAR (Accumulation Area Ratio) of about 0.8. For these parameters, based on the formula presented by Roe and Baker [42] and the melt factor and atmospheric lapse time given therein, as well as the mass balance determined by Sobota et al. [21], the response time is equal approximately 3.3 years. Thus, it supports the second option. However, it should be remembered that the parameters, which are correct of Mount Baker (Washington, DC, USA) glaciers do not have to be appropriate for the Ecology Glacier. To resolve this problem definitively, further investigation of range changes and balance mass is needed.

As already mentioned, the Ecology Glacier is characterized by a distinctively higher change in front retreat dynamics than Sphinx Glacier. This may be due not only to different morphologies, but also to the different thermal structures of the glaciers in their frontal zones. The mean ice temperature in this area at a depth of 10 m for the Ecology Glacier is −0.3 °C, whereas for Sphinx Glacier it is −1.0 °C [21]. The impact of the thermal structure on the difference in ice thickness changes is not visible in their size in the frontal zone. However, differences in glacial temperature cannot be ruled out as a cause of the different ranges of the thickness reduction zones relative to the front (see Table 3).

In numerous studies, climate changes are indicated as one of the main causes of glacier recession and progressing deglaciation. Battke et al. [22] suggested that the increase in the annual mean air temperature by 0.022 °C a^−1^ and the negative trend of total annual precipitation at the pace of 5.8 mm a^−1^ [19], resulting in a negative annual glacier mass balance, were the main reasons behind the decrease in glacier surface in ASPA No. 128 (formerly SSSI-8) over the period 1979–1999. Battke et al. [22] wrote that the ablation during the summer increased by approximately 20%, whereas the decrease in annual precipitation over 20 years also constituted about 20% of the mean annual long-term sum of the precipitation. This results in a fast increase in the amount of non-glaciated surfaces in this area. In 1979 the total land area was 17.89 km^2^, of which 14.91 km^2^ accounted for glaciated areas. In 1999 the total land area was 17.04 km^2^ of which only 7.98 km^2^ was covered with ice. Thus, the glaciated surface decreased by almost half over that 20-year period. A similar rate of total glacier surface reduction of Ecology and Sphinx Glaciers (41%) over a slightly longer period (1979–2012) was calculated in [21].

The results obtained in this paper confirm the increase in air temperature on King George Island between 1948 and 2016. However, the rate of warming has significantly slowed down because of a cool-down over the period 2012–2016. Between 1948 and 2016, the trend of the mean air temperature increase was equal to 0.015 °C a^−1^. For comparison, Kejna et al. [20] determined that between 1948 and 2011 this increase was 0.019 °C a^−1^. In contrast, precipitation data did not reveal any statistically significant trend (data from Bellingshausen station in the years 1968–2016). From a multiannual aspect, with comparable accumulation, increased ablation connected to the increase in air temperature can be observed, which in turn leads to a negative glacier mass balance and reduction of its range, surface, elevation, and volume. Precipitation is characterized by a significant variation on a yearly basis. The annual precipitation sum at Bellingshausen station was 500–1000 mm. Similarly, the mean annual air temperature on King George Island oscillates around the trend line, which has increased by about 1.0 °C since 1948. The combined influence of those fluctuations is the cause of the variable retreat rate observed in this paper for the studied glaciers. It should be noted that snowfall in the interior of the island is an especially important factor for the mass balance of the analyzed glaciers. Unfortunately, the data on this topic is not well documented.

## 6. Conclusions

The main conclusions which result from the complex research presented in this paper, including many multi-sourced and multi-temporal analyses, can be listed as follows:

1. In ASPA No. 128 during the period between 1956 and 2015, there was a significant recession of the glaciers’ fronts and a decrease in their thicknesses. Climate changes (mainly an increase in air temperature) are the primary cause of the observed glaciers’ recession and progressive deglaciation.
2. In the period 2001–2013, the glacier thickness reduction only occurred in the frontal zone of the glaciers and reached a maximum of approximately 30 m, irrespective of their different morphologies. In this area, the average annual reduction in elevation in this period was 1.5–2.3 m a^−1^ (for the Ecology Glacier and the northern part of Baranowski Glacier) and 0.8–2.3 m a^−1^ (for the southern part of the Baranowski Glacier and Sphinx Glacier).
3. The morphology of glaciers is a major element affecting the recession rate of their fronts. Acceleration of this rate is particularly dependent on the presence of the ice-cliff front because of the calving process which increase ablation. In the period 2001-2015, the Ecology Glacier and the northern part of the Baranowski Glacier are characterized by the ice-cliff front and have recession rates of 15–25 m a^−1^ and 10–20 m a^−1^, respectively. These rates are significantly higher than the rate of recession for the same period, estimated as 5–10 m a^−1^ for the southern part of the Baranowski Glacier and Sphinx Glacier, which are glaciers with fronts ending gently on the land.
4. More rapid acceleration of recession occurs when the front is in contact with water, because of the water’s additional impact on the ice melting at the base of the front, resulting in even more calving. This is confirmed by the difference between the rate of tidewater in Ecology Glacier’s recession, and the recession rate of the northern part of the Baranowski Glacier, which also has an ice-cliff front but ends on the land.
5. Despite the same size of the thickness reduction for all glaciers, the Ecology Glacier has a much larger thickness reduction zone, which also reaches much higher and further from the front. This is probably due to the different thermal structure of this glacier compared to others.
6. The longitudinal slope of the glaciers’ surfaces is the connector between the rate (and consequently size) of the glacier thickness changes and the changes of the glacier front position.
7. The dynamics of the studied glaciers are similar to other glaciers on King George Island. Changes in the front positions of small glaciers are an excellent indicator of climate changes with little delay. This study also confirms that the use of methods based on surveying and remote sensing data achieves results highly consistent with those obtained by standard glaciological methods.

## Figures and Tables

**Table 1:** Mean air temperature on King George Island (1948–2016) and mean annual precipitation at the Bellingshausen Station (1968–2016) in selected periods.

Period	Air Temperature (°C)	Precipitation (mm)
1948–1950	−3.6	
1951–1960	−2.8	
1961–1970	−2.7	
1971–1980	−2.7	687.2
1981–1990	−2.2	686.0
1991–2000	−2.1	719.5
2001–2010	−2.0	680.4
2011–2016	−2.5	693.6
Mean	−2.5	697.9

**Table 2:** Source data and application in the performed analyses.

Date of Survey or Photography	Type of Data and the Author	Type of Analysis
20 December 1956	FIDASE aerial photographs (British Government Ministry of Overseas Development)	border of the glaciers, DEM
9 February1979	aerial photographs (Institute of Ecology at Polish Academy of Science)	border of the glaciers
2001	measurement registered by surveying and GPS RTK technique (Rafał Pudełko)	border of the glaciers
2001	topographic map of Site of Special Scientific Interest No. 8 (SSSI-8) at scale of 1:12,500 (Rafał Pudełko)	DEM
2007	measurement registered by surveying and GPS RTK technique (Rafał Pudełko), orthophotomap of the Western shoreof the Admiralty Bay at scale of 1:10,000 (Rafał Pudełko)	border of the glaciers
2 February 2011	satellite images taken by GeoEye-1 satellite system	border of the glaciers
13 March 2013	satellite images taken by Pleiades-1A satellite system	border of the glaciers, DEM
21 January 2014	satellite images taken by GeoEye-1 satellite system	border of the glaciers
March 2015	terrestrial laser scanning and measurement using GNSS RTK technique (Maria Kowalska, Sławomir Łapiński, Mariusz Pasik, Marcin Rajner)	border of the glaciers

**Table 3:** The approximate values of the thickness reduction gradient components (longitudinal and vertical) for the analyzed glaciers for the period 2001–2013.

Name of Glacier.	Maximum Thickness Reduction in Frontal Zone [m]	Width of Thickness Reduction Zone [m]	Approximate Values of the Glacier Surface Slope (in 2013)	Thickness Reduction Gradientlong./vert.
Ecology Glacier	30	1000–1100	0.12	0.029/0.24
Baranowski Glacier (np)		600	0.10	0.050/0.50
Baranowski Glacier (sp)		800	0.11	0.038/0.35
Sphinx Glacier		300–600	0.16	0.067/0.42

sp—southern part, np—northern part.

**Table 4:** The results of the approximate analysis of the slope and ice thickness reduction effect on the size of the fronts’ recessions for the period 2001–2013 for analyzed glaciers.

Name of Glacier	Values of the Maximal Ice Thickness Reduction above Glacier Front in 2013 [m]	Approximate Values of the Glacier Surface Slope	Recession as the Slope and Ice Thickness Reduction Effect [m]	Observed Recession [m]
Ecology Glacier	20–30	0.12	170–250	250–400
Baranowski Glacier (np)	20–30	0.10	200–300	100–300
Baranowski Glacier (sp)	10–30	0.11	90–270	100
Sphinx Glacier (np)	10–30	0.20	50–150	50–100
Sphinx Glacier (sp)	10–30	0.10	100–300	50–100

sp—southern part, np—northern part.

## Data Availability

Archival aerial data available in a publicly accessible repository EarhExplorer.usgs.gov. The VHR satellite data are not publicly available due to licensing conditions.
